# Inconsistent definitions of transplant ineligibility in multiple myeloma: A systematic review

**DOI:** 10.1111/bjh.70323

**Published:** 2026-01-02

**Authors:** Karun Neupane, Mitch Singstock, Darshi Shah, Riyasha Dahal, Hira Mian, Febe Smith, Rajshekhar Chakraborty, Samer Al Hadidi, Maria Mainou, Ariel Grajales‐Cruz, Syeda Mahrukh Hussnain Naqvi, Kenneth H. Shain, Aaron Goodman, Luciano J. Costa, Muzaffar Qazilbash, Ghulam Rehman Mohyuddin

**Affiliations:** ^1^ Department of Hematology and Oncology Moffitt Cancer Center, University of South Florida Tampa Florida USA; ^2^ Department of Internal Medicine University of Utah Salt Lake City Utah USA; ^3^ Renaissance School of Medicine at Stony Brook University Stony Brook New York USA; ^4^ Department of Internal Medicine Manatee Memorial Hospital Bradenton Florida USA; ^5^ McMaster University Hamilton Ontario Canada; ^6^ Amsterdam University Medical Center Amsterdam The Netherlands; ^7^ Columbia University Medical Center New York New York USA; ^8^ University of Texas Southwestern Medical Center Dallas Texas USA; ^9^ Clinical Research and Evidence Based Medicine Unit Aristotle University of Thessaloniki Thessaloniki Greece; ^10^ Department of Biostatistics and Bioinformatics, Moffitt Cancer Center University of South Florida Tampa Florida USA; ^11^ MountainView Hospital Las Vegas Nevada USA; ^12^ O'Neal Comprehensive Cancer Center, Department of Hematology and Oncology University of Alabama Birmingham Alabama USA; ^13^ MD Anderson Cancer Center Houston Texas USA; ^14^ Division of Hematology Huntsman Cancer Institute, University of Utah Salt Lake City Utah USA

**Keywords:** autologous stem cell transplant, frailty, myeloma, newly diagnosed multiple myeloma, transplant ineligibility

## Abstract

High‐dose melphalan followed by autologous stem cell transplantation (ASCT) is the standard of care for eligible patients with newly diagnosed multiple myeloma (NDMM). A substantial proportion of patients are deemed ineligible for ASCT due to age, comorbidities, performance status and/or frailty. Criteria defining transplant ineligibility remain inconsistent and poorly characterized. We conducted a systematic review that assessed phase II, III and IV randomized controlled trials (RCTs) through March 2025. We included a total of 55 studies that enrolled transplant‐ineligible/deferred NDMM patients. Among 55 transplant‐ineligible trials, only 47% explicitly defined ineligibility criteria. Of these, 44% of trials used age as a cut‐off with/without other criteria. Only two studies explicitly specified which comorbidities constituted transplant ineligibility. When age was utilized as a cut‐off, age ≥65 was the most commonly used cut‐off. The median age of participants in these trials ranged from 62 years to 78.5 years and showed a trend upwards over time (*p* = 0.1388). Performance status of enrolled patients was reported inconsistently. Frailty tools were reported in 22% of studies. RCTs enrolling transplant‐ineligible patients with NDMM demonstrated considerable heterogeneity in defining ineligibility. While the decision to pursue ASCT remains individualized, the absence of evidence‐based definitions of transplant ineligibility complicates research interpretation and clinical decision‐making.

## Graphical abstract

This systematic review of 55 trials reveals significant inconsistency in defining transplant ineligibility for multiple myeloma. Arbitrary age cut‐offs predominantly drive exclusion, while validated frailty tools and specific comorbidities are frequently underutilized. Standardized, evidence‐based criteria are essential to optimize patient selection and improve clinical decision‐making.

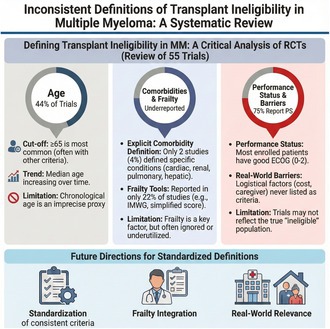

## INTRODUCTION

The standard treatment paradigm for transplant‐eligible patients with newly diagnosed multiple myeloma (NDMM) consists of induction therapy followed by high‐dose melphalan and autologous stem cell transplantation (ASCT) as consolidation therapy. In contrast, transplant‐ineligible patients receive extended multi‐agent therapy regimens, though historically, the most durable remissions have been achieved through treatment approaches incorporating upfront ASCT.^1^, ^2^

Advances in supportive care have significantly expanded the feasibility of ASCT, resulting in a sevenfold increase in ASCT over the past three decades.^3^ Despite these improvements, a substantial proportion of patients with NDMM remain ineligible for transplantation due to advanced age, significant comorbidities, poor functional status, frailty or compromised Eastern Cooperative Oncology Group (ECOG) performance status. The determination of transplant eligibility in multiple myeloma involves considerable inter‐institutional variability and subjective clinical judgement, yet no systematic analysis has comprehensively characterized the criteria used to define transplant ineligibility.^4^

An additional category of patients, those who are transplant‐deferred, presents unique clinical considerations. These patients may be potentially eligible for ASCT but face delays in treatment due to factors such as patient preference, logistical constraints or physician choice. Recent clinical trials, including the CEPHEUS study, have enrolled such transplant‐deferred patients. The promising outcomes seen in these trials require consideration of distinct approaches that differ from the historical approach of transplant‐eligible or ineligible cohorts.^5^

No prior study has ever evaluated how transplant ineligibility is defined in a cohort of NDMM trials. The purpose of this study was to assess how transplant ineligibility is defined and to identify the characteristics of trials that enroll transplant‐ineligible patients.

## METHODS

Direct patient data were not obtained, and all the information was gathered from publicly available and de‐identified sources; therefore, this study was considered exempt from institutional review board approval.

### Initial search strategy

**FIGURE 1:**
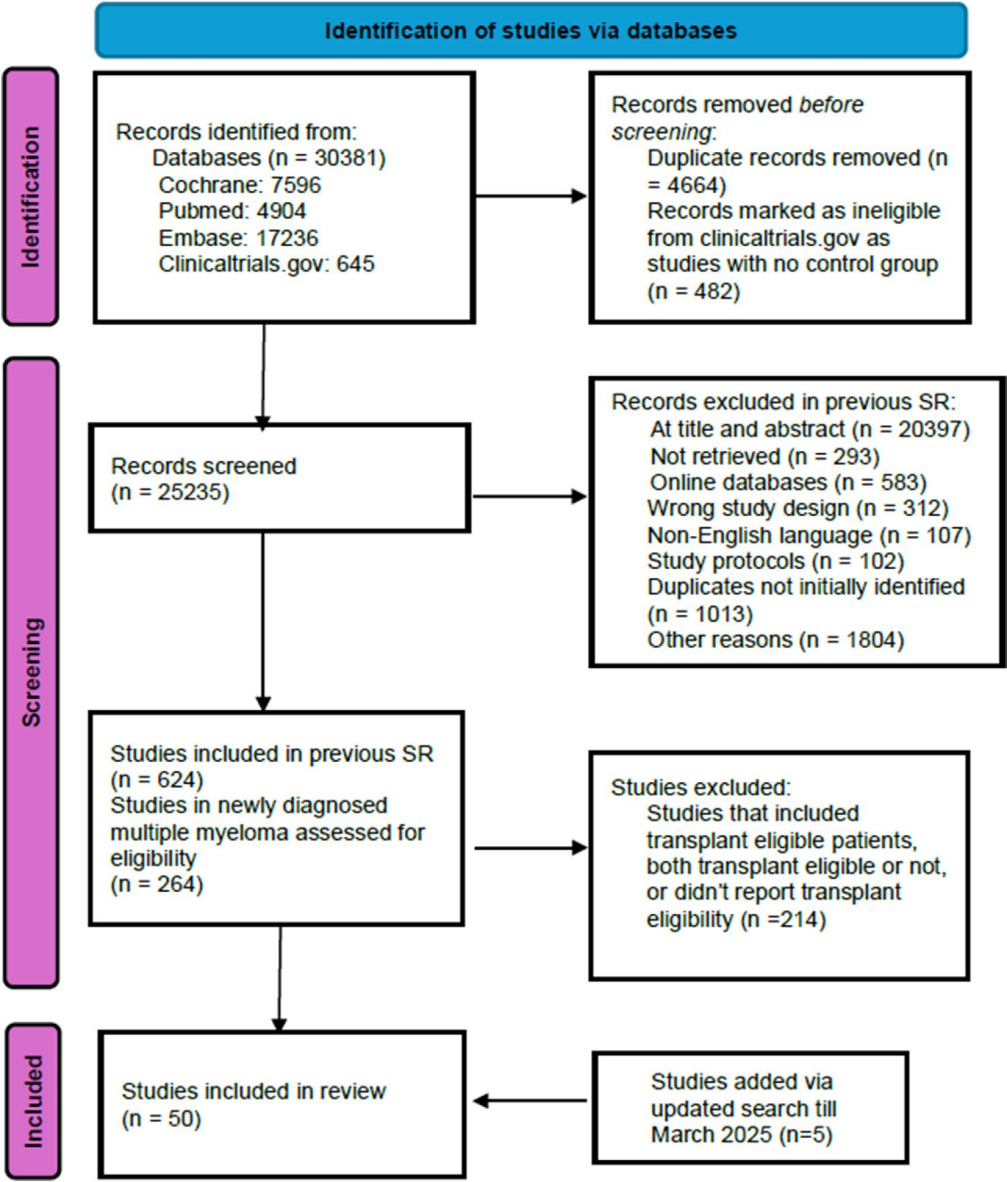
PRISMA flow diagram depicting our study selection strategy for this review.

A comprehensive search was performed across four databases: (MEDLINE/PubMed, Embase, Clinicaltrials.gov and Cochrane Registry of RCTs). Detailed search terms are provided in the Supplement. Two independent reviewers (KN and MS) screened all studies, with any conflicts resolved through mutual discussion. The systematic review was conducted in accordance with the Preferred Reporting Items for Systematic Reviews and Meta‐Analyses (PRISMA) guidelines (Figure 1).^6^

The original search strategy was restricted to myeloma RCTs published in peer‐reviewed journals till October 2023. The search was later updated to include studies published through March 2025. Studies were excluded if they were editorials, case reports, case series, review articles, case–control studies, retrospective/prospective cohort studies, single‐arm studies or non‐English language publications. Conference abstracts captured through database searches were included in the final analysis. As this represents a secondary analysis of a previously conducted systematic review, separate PROSPERO registration was not required. The original search strategy is publicly accessible at https://osf.io/mxk8t.^7^, ^8^

### Selection of studies and data extraction

After initial screening of RCTs in myeloma, we included only those studies that specified the enrolment of transplant‐ineligible NDMM patients, either in the title or in the methods section of the publication. Studies that enrolled transplant‐ineligible and transplant‐deferred patients like CEPHEUS^9^ were also included. To capture additional information on frailty assessments, we conducted a secondary search using a snowball methodology to identify any separate publications related to the included trials that reported on frailty‐specific outcomes.^10^ Further information pertaining to the selection of studies and data extraction is in the Supporting Information.

The primary outcome of this study was to assess how transplant ineligibility criteria are defined in myeloma RCTs. Secondary outcomes included evaluation of frailty reporting practices in these trials and temporal analysis of median age trends among enrolled patients.

### Statistical analysis

The statistical methods and analyses used in the study have been summarized in Table S1.

## RESULTS

Of the 264 RCTs reviewed, 50 (19%) enrolled transplant‐ineligible/deferred patients with NDMM. An updated search identified five additional studies, bringing the total number of trials included to 55. Among these, 13 (24%) were phase II RCTs, 39 (71%) were phase III and 1 (2%) was phase IV. Forty‐nine trials (89%) were available as full manuscripts, while 6 (11%) were available as abstracts only.

### Information added from full protocols

Study protocol/supplementary data were available for 35 studies (64%) either as supplementary material in the primary publication or via trial registration records on ClinicalTrials.gov. Based on information obtained from the protocol and supplementary materials, we identified and added transplant ineligibility criteria for three studies (6%) that were not reported in the primary manuscript.^11^, ^12^, ^13^

### Geographical distribution and timeline of studies

Majority of the studies (*n* = 39) (71%) were limited to a single geographical region, that is, Asia, Europe, South America or Australia; 12 (22%) were multiregional while 4 (7%) were conducted in the United States only. The number of studies including only transplant‐ineligible myeloma increased through the years with only 3 (5%) reported between 1996 and 2005, 19 (35%) reported between 2006 and 2015 and 33 (60%) reported between 2016 and 2025.

### Reasons for transplant ineligibility

Among 55 transplant‐ineligible RCTs, only 26 (47%) explicitly defined ineligibility criteria as depicted in Table 1. Of these, 24 (44%) trials used age as a cut‐off with/without other criteria, combination of age/comorbidities (*n* = 18, 33%) being the most common criteria. Notably, only two studies (4%)^12^, ^13^ explicitly defined the comorbidities that rendered patients ineligible for transplant, specifying cardiac, renal, pulmonary or hepatic dysfunction as qualifying conditions. Of these, only one study provided further detail, defining organ dysfunction as: left ventricular ejection fraction (LVEF) <40%, forced expiratory volume in 1 s (FEV_1_) <40%, bilirubin >1.5 times the upper normal limit (UNL), AST/ALT >2.5 × UNL and creatinine clearance <60 mL/min.^13^

**TABLE 1:** Definition of transplant ineligibility.

Definition of transplant ineligibility	Number of trials (*n*)	% of all trials (*N* = 55)	References
Age alone	2	4%	[14, 15]
Age + comorbidities	18	33%	—
Age + comorbidities + patient preference	2	4%	[13, 16]
Age + comorbidities + insufficient stem cells + Patient preference	1	2%	[17]
Age + No access (cost or other reasons) + patient preference	1	2%	[11]
Investigator judgement	1	2%	[18]
Comorbidities + patient preference	1	2%	[19, 20]

### Age characteristics in transplant‐ineligible trials

Twenty‐four (44%) trials used age cut‐offs for transplant ineligibility. Among them, 22 (40%) used an age cut‐off of 65 or more while the remaining 2 (4%) used an age cut‐off of 70.^9^, ^21^ A total of 49 trials (90%) reported the median age of enrolled patients. This ranged from 62 to 78.5 years.

**FIGURE 2:**
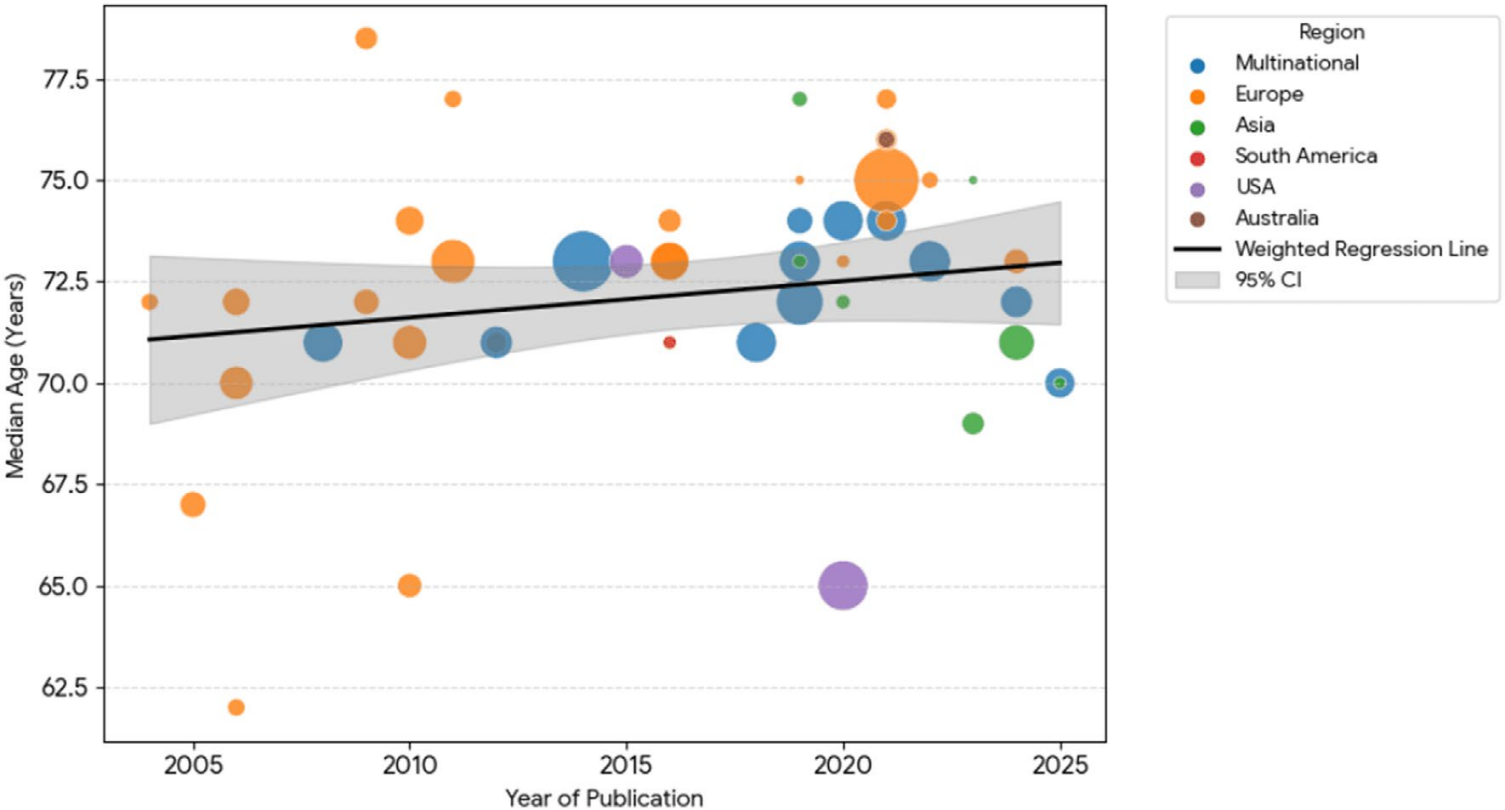
Weighted linear regression plot suggesting an increasing trend in median age of transplant‐ineligible NDMM patients enrolled in trials over time that does not reach statistical significance. Each grey dot represents the observed median age from an individual clinical trial. NDMM, newly diagnosed multiple myeloma.

The median age in these trials showed an upward trend over time, as indicated by Spearman correlation (*ρ* = 0.219, *p* = 0.1388), though this was not statistically significant. A weighted linear regression, accounting for study sample size, estimated an average annual increase in median age of 0.09 years (95% CI: −0.06 to 0.24, *p* = 0.236); this trend also did not reach statistical significance (Figure 2). Thirty‐seven trials (67%) included patients of age 80 or more.

### Performance status characteristics in transplant‐ineligible trials

While none of the trials specified performance status as an ineligibility criterion for transplant, 41 (75%) trials reported performance status as an inclusion criterion for the study. Seventeen (31%) of them restricted inclusion criteria to patients with ECOG performance status of 0–2. Two trials (4%) included patients with an ECOG performance status of 0–1,^12^, ^22^ 10 trials (18%) included patients with ECOG performance status of 0–3 and nine trials (18%) included patients with ECOG performance status of 0–4. In those trials, patients with ECOG performance status 3–4 represented a small minority ranging from 1% to 26%. Six trials (11%) used Karnofsky performance score (KPS) and included patients with KPS >60% (*n* = 5)^14^, ^23^, ^24^, ^25^, ^26^ and >50% (*n* = 1).^16^

### Use of frailty tools

A minority of trials (*n* = 12, 22%) reported use of frailty tools. This includes three studies (6%) identified via snowball method that had separate publications to report frailty.^27^, ^28^, ^29^ The utilized frailty tools include International Myeloma Working Group frailty score (*n* = 8, 15%) and simplified frailty score (*n* = 4) (7%). Of these 12 studies, one trial (2%) enrolled only frail patients^30^ while one trial completely (2%) excluded frail patients.^31^ The remaining 10 trials (22%) included frail patients ranging from 24% to 54% of the study population as follows: 24%,^32^ 25%,^33^ 26%,^34^ 40%,^13^, ^35^ 44%,^36^ 45%,^37^ 46%,^38^ 49%^29^ and 54%.^28^

### Use of non‐medical reasons as a criterion for transplant ineligibility

Only one trial reported lack of access due to cost or other reasons as a criteria for transplant ineligibility but did not further specify those other reasons.^11^

## DISCUSSION

In this systematic review evaluating how transplant ineligibility is defined in myeloma RCTs, we found considerable inconsistency in the definitions used, with many studies relying on arbitrary age cut‐offs rather than frailty or other evidence‐based criteria. Frailty tools were inconsistently utilized, and the exact comorbidities that defined one as transplant ineligible were not specified. Furthermore, common barriers to transplant in the real world, such as logistical reasons or unavailability of caregivers,^39^ were never listed as exclusionary criteria for transplant in clinical trials.

We found that the median age of enrolled patients in these trials showed a trend towards increasing, reflecting increased physician and patient comfort level with transplanting older patients even though the definition of transplant ineligibility and age cut‐offs as listed in inclusion criteria did not change. Another factor contributing to increasing age in these trials could also be the inclusion of otherwise eligible patients in the ‘transplant‐deferred’ category. We found that, even in these transplant‐ineligible trials, most patients enrolled had an ECOG performance of 0–2, the majority of patients were not frail and patients with poor performance status were excluded from those trials.

Although ASCT continues to improve the duration of remission and progression‐free survival, recent trials have not yet shown an overall survival benefit for these interventions.^40^, ^41^ More recently, excellent outcomes have been observed even without the use of ASCT, such as in the IMROZ and CEPHEUS trials.^5^, ^42^ Of note, the CEPHEUS trial excluded patients who were frail, and no enrolled patients were above the age of 80, while the IMROZ trial explicitly excluded patients age greater than 80. The MIDAS trial demonstrated that, in patients who had already achieved measurable residual disease (MRD) negativity at 10^−5^ with quadruplet induction therapy, consolidation with either ASCT or continued quadruplet therapy (isatuximab, carfilzomib, lenalidomide, dexamethasone) resulted in similarly high rates of MRD negativity at 10^−6^, questioning the added benefit of ASCT in patients who achieve MRD negativity after induction.^43^ However, these trials have had relatively limited follow‐up durations, and longer term outcomes beyond 5 years remain to be established. Additionally, the median follow‐up periods in these studies may be insufficient to fully capture late relapses or assess the durability of responses.

In countries with limited access to other therapies, transplant remains an integral and cost‐effective tool through which remission can be lengthened.^44^ Globally, outcomes after ASCT are favourable, with non‐relapse mortality (NRM) rates of 1%–3% depending on region, but there is substantial variability in transplant rates and outcomes due to differences in patient selection, resource availability and use of maintenance therapy.^45^ In Asia, resource‐stratified guidelines recommend ASCT for fit patients under 65–70 years in centres with adequate resources, but access remains limited in some settings, and age cut‐offs may be more strictly applied.^46^ In North America and much of Europe, ASCT remains the standard of care for eligible patients, with increasing utilization among older adults.^3^ Non‐relapse mortality following ASCT remains very rare, even in cohorts of patients above the age of 75, highlighting how arbitrary age cut‐offs may not be suitable to determine transplant candidacy.^47^ In a study by Mizuno et al., the 3‐year OS rates among NDMM patients who underwent ASCT were 85.9% for ages 18–39, 82.8% for 40–64, 81.1% for 65–69, 78.4% for 70–74 and 74.8% for those ≥75 years, with NRM remaining very low across all age groups. Importantly, the 3‐year cumulative excess mortality was similar between age cohorts (ranging from 13.1% to 15.0%), indicating that advanced age alone does not substantially increase post‐transplant risk and should not preclude ASCT in appropriately selected older adults.^48^ Moreover, the substantially higher NRM rates currently observed with newer therapeutic modalities such as ciltacabtagene autoleucel (cilta‐cel) raise questions about the clinical rationale for classifying patients as ineligible for transplantation yet suitable candidates for cilta‐cel therapy, as is being done in ongoing trials (NCT04923893, CARTITUDE‐5).

Despite the availability of validated frailty scores such as the IMWG frailty index and other simplified scores, frailty remains underreported and inconsistently assessed in clinical trials for myeloma.^29^, ^49^ This omission is particularly concerning given that the transplant‐ineligible population is often older and more physiologically vulnerable, with frailty significantly influencing treatment tolerability, toxicity and survival outcomes.^49^

Our study has several limitations. We restricted our analysis to randomized controlled trials, which may have excluded valuable data from real‐world studies or retrospective analyses. Moreover, despite implementing a structured search strategy, it is possible that some relevant trials were missed due to limitations in indexing, variations in terminology used to describe transplant ineligibility or incomplete trial registration. Another important limitation is the lack of access to full trial protocols for all the studies, which hindered our ability to comprehensively evaluate how transplant ineligibility was defined across studies. This includes the potential omission of key eligibility criteria such as frailty, comorbidities or performance status that may have influenced trial enrolment but were not explicitly reported. These limitations may have led to an underappreciation of the heterogeneity in defining transplant ineligibility and highlight the need for more transparent and standardized reporting in future clinical trials. Due to a limited number of trials that enrolled only in the United States, regional differences in how transplant ineligibility were defined were unable to be ascertained.

As a result of arbitrary age cut‐offs, patients with identical clinical profiles may receive fundamentally different treatment approaches based solely on where they seek care or which clinical trial they might access. There is no biological reason why a 65‐year‐old patient should be treated differently than a 64‐year‐old patient. Chronological age is an imprecise proxy for physiological reserve and does not reliably reflect functional status, organ function or tolerance to intensive therapy.^50^ A recent population‐based study in Ontario showed a pronounced decline in ASCT utilization with increasing age.^51^ Even after adjusting for comorbidities and performance status, older patients were significantly less likely to receive ASCT, suggesting that age alone continues to unduly influence treatment decisions. In contrast, frailty assessments incorporate a multidimensional view of a patient's health, offering a more accurate basis for determining fitness for transplant and optimizing outcomes.^52^

In summary, we find that, in myeloma RCTs, transplant ineligibility is defined arbitrarily, often based on age 65, and that most patients enrolled in transplant‐ineligible trials are not frail. Although the decision on whether to pursue transplant is a highly nuanced one, the current definitions of eligibility for transplant remain arbitrary and inconsistent, with consequences both for individual patients enrolled on these trials and for clinical decision‐making in the real world.

## AUTHOR CONTRIBUTIONS

G.R.M. conceived the research idea. M.M. performed an initial search. M.S., K.N. and R.D. screened the studies and collected data. KN and GRM performed data analysis. G.R.M., K.N. and D.S. wrote the first draft of the manuscript. S.M.H.N. conducted statistical analysis. All authors provided critical input on the manuscript, the methodology of the study, reviewed the initial draft of the manuscript and approved the final version of the manuscript.

## FUNDING INFORMATION

This study did not receive any funding.

## CONFLICT OF INTEREST STATEMENT

K.N.: Research funding from Moffitt Cancer Center's Junior Scientist Research Partnership Grant and Conquer Cancer ASCO Foundation. M.S.: Research funding from Pfizer and AstraZeneca for prostate cancer research. D.S.: No disclosures. R.D.: No disclosures. H.M.: Honoraria/Consultancy: Janssen, BMS, Takeda, Amgen, Forus, Pfizer; Research funding: Janssen, Pfizer. F.S.: No disclosures. R.C.: Honoraria: Guidepoint Global; Consulting/Advisory: Janssen, Sanofi Pasteur, Adaptive Biotechnologies; Research funding: Genentech. S.A.H.: Research funding: Pfizer, Janssen; Consulting: Janssen, Sanofi. M.M.: No disclosures. A.G.‐C.: Consulting/Advisory: Cellectar, Sanofi; Speakers' Bureau: Amgen, Sanofi. S.M.H.N.: No disclosures. K.H.S.: Advisor/Consultant: Celgene, Janssen, Takeda, Bristol‐Myers Squibb; Research funding: AbbVie. A.G.: Consultancy: Seattle Genetics. L.J.C.: Honoraria: AbbVie, Adaptive Biotechnologies, Amgen, Celgene, Janssen, Karyopharm Therapeutics, Sanofi; Consulting/Advisory: AbbVie, Adaptive Biotechnologies, Amgen, Bristol‐Myers Squibb/Celgene, Janssen, Pfizer, Sanofi; Speakers' Bureau: Amgen, Sanofi; Research funding: Amgen, Bristol‐Myers Squibb/Celgene/Juno, Caribou Biosciences (Inst), Janssen. M.Q.: Advisory board: Oncopeptides; Data Safety Monitoring Board: Autolus. G.R.M.: Honoraria: MashupMD, Medscape (writing); Institutional research funding: Janssen (site PI role).

## Supporting information


Data S1.

## Data Availability

Data can be shared upon a reasonable request to the corresponding author.
