# Supplementary material for: Inconsistent definitions of transplant ineligibility in multiple myeloma: A systematic review
Source: Br J Haematol. 2026 Jan 2;208(3):829–36. doi: 10.1111/bjh.70323 (PMC12995538; doi:10.1111/bjh.70323)
Supplement: Supplementary file 1 — Data S1. [file BJH-208-829-s001.docx]

# Selection of studies and data extraction:

We reviewed the protocol of each trial, when available, either as supplementary material in the primary publication or via trial registration records on ClinicalTrials.gov. This allowed us to extract detailed information on transplant ineligibility criteria that may not have been explicitly stated in the main manuscript. Three authors (KN, MS, and RD) performed and verified all data extraction. Extracted data was tabulated using Microsoft Excel (Microsoft, Redmond, Washington, United States). We identified the following characteristics of studies: name of the RCT, year of publication, type of publication, location of study, number of participants, transplant eligibility of participants, median age, age range, performance status of patients, frailty, and criteria for transplant ineligibility.

# Search strategy:

## PUBMED:

(((("Multiple Myeloma"[Mesh]) OR "Plasmacytoma"[Mesh]) OR (multiple myeloma OR plasmacytoma OR plasmacytom* OR myelom*))) AND ((randomized controlled trial[pt] OR controlled clinical trial[pt] OR randomized[tiab] OR placebo[tiab] OR clinical trials as topic[mesh:noexp] OR randomly[tiab] OR trial[ti] NOT (animals[mh] NOT humans [mh])))

## COCHRANE: (limited to trials)

MeSH descriptor: [Multiple Myeloma] explode all trees MeSH descriptor: [Plasma Cells] explode all trees

Multiple myeloma:ti,ab,kw Plasmatocytoma:ti,ab,kw Plasmatocytom*:ti,ab,kw Myelom*:ti,ab,kw

## EMBASE:

Ab(Multiple Myeloma[Mesh]) OR ab(“multiple myeloma”) OR ab(plasmacytoma*) OR ab(myelom*) OR ab(Plasmacytoma [Mesh])

AND (su.exact.explode(“clinical trial” OR “clinical trial (topic)” OR “clinical trials as topic”) OR qu(“clinical trial”) OR dtype,ti,su,subst(“clinical trial” OR “clinical trials” OR “clin trial” OR “equivalence trial” OR “equivalence trials” OR “multicenter study” OR “multicenter studies” OR “randomized controlled trial” OR “randomized controlled trials”))

AND ((human OR humans OR man OR men OR women OR woman OR patient OR patients OR volunteer OR volunteers OR “homo sapiens” OR Hominidae OR male OR males OR female OR females OR adult OR adults))

# Supplementary table:

Supplementary Table 1 Statistical methods and analyses used in this study.

| Statistical Method | Description |
| --- | --- |
| Descriptive Statistics | Used to summarize study characteristics, including median age, sample size, and year of publication. |
| Categorical Variables | Reported as frequencies and percentages. |
| Continuous Variables | Summarized using mean, median, standard deviation, minimum, and maximum. |
| Correlation Analysis | Spearman rank correlation was used to assess the association between median age and year of publication. |
| Trend Analysis | A weighted linear regression model (PROC GLM) (50) was fitted with year of publication as the independent variable and median age as the dependent variable. Study sample size was used as weight. |
| Model Diagnostics | Model assumptions (linearity, homoscedasticity, normality of residuals) were evaluated using residual diagnostic and Q-Q plots (50). Analyses were performed using SAS version 9.4 (SAS Institute, Cary, NC, USA). |

Reference:

50. Inc. SI. SAS/STAT® 14.1 User’s Guide The GLMProcedure 2015 [Available from: <https://support.sas.com/documentation/onlinedoc/stat/141/glm.pdf>.
